# Estimating the probabilities of rare arrhythmic events in multiscale computational models of cardiac cells and tissue

**DOI:** 10.1371/journal.pcbi.1005783

**Published:** 2017-11-16

**Authors:** Mark A. Walker, Viatcheslav Gurev, John J. Rice, Joseph L. Greenstein, Raimond L. Winslow

**Affiliations:** 1 Department of Biomedical Engineering and Institute for Computational Medicine, Johns Hopkins University, Baltimore, MD, United States of America; 2 TJ Watson Research Center, IBM, Yorktown Heights, NY, United States of America; Universiteit Gent, BELGIUM

## Abstract

Ectopic heartbeats can trigger reentrant arrhythmias, leading to ventricular fibrillation and sudden cardiac death. Such events have been attributed to perturbed Ca^2+^ handling in cardiac myocytes leading to spontaneous Ca^2+^ release and delayed afterdepolarizations (DADs). However, the ways in which perturbation of specific molecular mechanisms alters the probability of ectopic beats is not understood. We present a multiscale model of cardiac tissue incorporating a biophysically detailed three-dimensional model of the ventricular myocyte. This model reproduces realistic Ca^2+^ waves and DADs driven by stochastic Ca^2+^ release channel (RyR) gating and is used to study mechanisms of DAD variability. In agreement with previous experimental and modeling studies, key factors influencing the distribution of DAD amplitude and timing include cytosolic and sarcoplasmic reticulum Ca^2+^ concentrations, inwardly rectifying potassium current (I_K1_) density, and gap junction conductance. The cardiac tissue model is used to investigate how random RyR gating gives rise to probabilistic triggered activity in a one-dimensional myocyte tissue model. A novel spatial-average filtering method for estimating the probability of extreme (i.e. rare, high-amplitude) stochastic events from a limited set of spontaneous Ca^2+^ release profiles is presented. These events occur when randomly organized clusters of cells exhibit synchronized, high amplitude Ca^2+^ release flux. It is shown how reduced I_K1_ density and gap junction coupling, as observed in heart failure, increase the probability of extreme DADs by multiple orders of magnitude. This method enables prediction of arrhythmia likelihood and its modulation by alterations of other cellular mechanisms.

## Author summary

Arrhythmias are electrical abnormalities of the heart that can degenerate into fibrillation, thus preventing normal heartbeats and leading to sudden cardiac death. The mechanisms leading to ventricular arrhythmias and the unexpected nature of sudden cardiac death are not fully understood. One hypothesis is that a group of cardiac myocytes, which generate contraction, spontaneously depolarize at precisely the same moment to excite the surrounding tissue. In individual myocytes, such misfires, known as delayed afterdepolarizations, are driven by random ion channel gating and thus stochastic in nature. While incidental afterdepolarizations in a large number of myocytes is highly improbable on any given beat, it may be feasible over a long time frame, thus explaining the unpredictability of arrhythmias. We developed a detailed model spanning the molecular, cellular, and tissue scales that realistically reproduces the mechanisms underlying this hypothesis. An efficient method is presented for estimating the probability of extremely rare delayed afterdepolarizations in tissue from a limited set of simulations. Furthermore, we demonstrate how altered tissue and ion channel properties in heart disease increase the risk of arrhythmia. This approach can be used generally to probe the effects of specific molecular mechanisms on the likelihood of rare delayed afterdepolarizations.

## Introduction

In cardiac myocytes, dyads are sites where the junctional sarcoplasmic reticulum (JSR) membrane closely approaches (~ 15 nm) invaginations of the cell membrane known as transverse tubules (TTs). Voltage-sensitive L-type calcium (Ca^2+^) channels (LCCs) are preferentially localized to the TT membrane of the dyad, where they closely appose Ca^2+^-binding Ca^2+^-release channels known as ryanodine receptors (RyRs) in the dyad JSR membrane. Depolarization of the cell membrane during an action potential (AP) increases LCC open probability, generating a flux of Ca^2+^ ions into the dyad. The resulting local increases of dyad Ca^2+^ concentration ([Ca^2+^]_d_) increase RyR open probability, which when open allow flux of Ca^2+^ ions from the JSR into the dyad. This process, known as Ca^2+^-induced Ca^2+^ release (CICR), causes brief, spatially localized release events known as a Ca^2+^ sparks [1]. Due to their synchronization, these Ca^2+^ sparks cause a cell-wide rise in cytosolic [Ca^2+^] ([Ca^2+^]_i_), leading to myofilament activation and force generation. This process, known as excitation-contraction coupling (ECC), is central to the function of the myocyte [2].

Ca^2+^ sparks can also occur randomly at a single release site when the spontaneous opening of a single RyR triggers the CICR process [3]. Under conditions promoting cellular Ca^2+^ overload, Ca^2+^ sparks are more likely to trigger RyRs at nearby release sites, thereby generating propagating Ca^2+^ waves [4]. Spontaneous Ca^2+^ release events generate an inward current via the Na^+^/Ca^2+^ exchanger (NCX), which transports 3 Na^+^ ions into the cell for every Ca^2+^ ion extruded, and additionally in canine myocytes via the Ca^2+^-activated chloride channel [5]. In diastole, this produces a net inward current, resulting in an elevation of cell membrane potential (V) known as a delayed afterdepolarization (DAD) [6]. DADs of sufficient amplitude can lead to the activation of the fast inward Na^+^ current (I_Na_) and trigger a premature AP. Gap junctions joining adjacent cells then conduct the aberrant AP across the myocardial syncytium. Such ectopic events in the heart can induce reentrant ventricular arrhythmias that lead to sudden cardiac death [7]. Furthermore, the propensity for spontaneous Ca^2+^ release is increased in heart diseases such as heart failure, hypertrophy, and some forms of long-QT syndrome, which are associated with increased risk for sudden cardiac death. Therefore, understanding Ca^2+^ dynamics in ventricular myocytes and the Ca^2+^ handling instability that arises under pathological conditions is fundamental to our understanding of cardiac arrhythmogenesis.

Experimental studies have observed triggered activity under conditions evoking spontaneous Ca^2+^ release in myocardial wedge preparations [8] and whole heart [9, 10]. These studies show that the likelihood of observing ectopic foci is correlated with the degree of Ca^2+^ loading. In isolated myocytes, Ca^2+^ waves are observed when the sarcoplasmic reticulum (SR) Ca^2+^ load achieves a critical level [11]. However, electrotonic coupling in tissue attenuates DAD amplitude by diverting inward current to adjacent myocytes through the gap junctions. Wasserstrom et al. reported that with increasing SR Ca^2+^ load, spontaneous Ca^2+^ waves exhibited greater synchrony following cessation of rapid pacing in intact heart [10]. Synchronous DADs result in smaller spatial gradients in membrane potential, less loss of depolarizing current into neighboring cells, and therefore larger DAD amplitude.

An elegant theoretical study by Chen et al. analytically investigated the probability of triggered events in a 1D fiber as a first passage time problem using a minimal model of Ca^2+^ release and membrane currents [12]. They showed that the expected time to a triggered event decreased according to a power law of the number of cells in the fiber, and that this effect depended on the balance of gap junction, NCX, and inwardly-rectifying potassium current (I_K1_) conductance. However, to be tractable, the model employed simplified models of the membrane currents. Here we present an approach that retains mechanistically realistic characterization of membrane current properties while at the same time is computationally tractable, permitting the evaluation of a sufficiently large number of stochastic simulations to estimate rare event probabilities.

In this study, we present a multicellular model of cardiac tissue which incorporates a stochastic biophysically detailed spatial model of the ventricular myocyte as its fundamental building block. The single cell model is used to study the roles of stochastic RyR gating and Ca^2+^ wave dynamics on the statistical distribution of DADs under pathophysiological conditions. The cellular Ca^2+^ load and density of I_K1_ are shown to be two important factors influencing the mean and variance of DAD amplitude and timing. We then develop a one-dimensional (1D) myocyte tissue model comprised of these cells, and present a method for estimating the probability of rare events that are “surrogates” for ectopic beats. These surrogate events are defined as the occurrence of (rare) high-amplitude DADs with membrane potential exceeding a threshold value V_T_. We develop a computationally efficient approach for estimating the probability of these threshold crossings. This method enables us to estimate how changes in model parameters influence the probability of these surrogate events. As an example, the effects of reductions of I_K1_ density and gap junction coupling on the distribution of DAD amplitude and therefore the probability of threshold crossings are demonstrated, providing quantitative insight into how electrophysiological remodeling affects the probability of potentially arrhythmogenic ectopic beats.

## Methods

### Ventricular myocyte model

**Fig 1:**
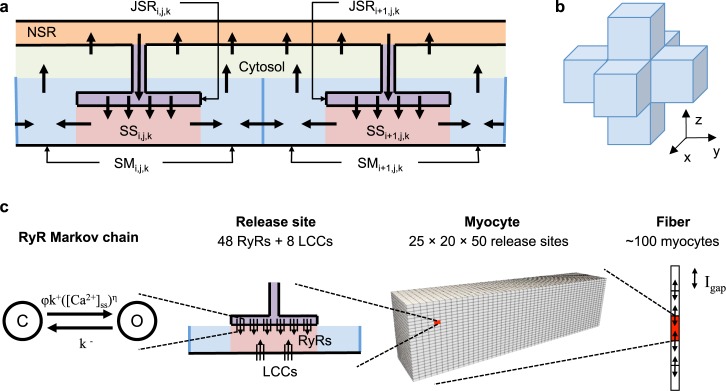
Multiscale cell and tissue model schematics. (a) Diagram of intracellular Ca^2+^ compartments and transport. At each release site at coordinates (i,j,k), Ca^2+^ is released via RyRs from the JSR into the dyadic subspace (SS) and diffuses into a submembrane (SM) compartment. Ca^2+^ diffuses between SM compartments of adjacent release sites in the 3D lattice, as depicted in panel (b). Ca^2+^ can also diffuse from the SM into a single cell-averaged cytosolic compartment and can be transported by the SR Ca^2+^ ATPase (SERCA pump) from the cytosolic compartment into a single network SR (NSR), which refills the JSR. (c) Illustration of the different spatial scales incorporated in the tissue model including (from left to right) the 48 stochastic RyRs in each of the 25,000 release sites of a single cell. Hundreds of cells are coupled via gap junction currents to form the fiber model.

We have developed a three-dimensional (3D) spatial model of a single myocyte based on the (non-spatial) Greenstein-Winslow canine ventricular myocyte model [13]. To enable reproduction of realistic Ca^2+^ waves and DADs, the original model was adapted to include spatial Ca^2+^ diffusion on a rectangular lattice of 25,000 Ca^2+^ release sites (Fig 1) distributed within the cell. The cell was divided into 25 and 20 lattice points in the two transverse directions and 50 lattice points in the longitudinal direction. Release sites were spaced 1 and 2 μm in the transverse and longitudinal directions, respectively [14]. The time constant for longitudinal Ca^2+^ diffusion was twice (i.e. 2x slower) that for the transverse direction such that the model exhibited symmetric Ca^2+^ wave propagation [15]. This difference in diffusion rates arises from differences in diffusion along versus across TTs.

A sub-membrane (SM) release site compartment (Fig 1A) was added to describe the volume under the TT membrane where the Ca^2+^ concentration ([Ca^2+^]) is elevated during Ca^2+^ sparks and cell-wide Ca^2+^ release [16]. Detailed imaging studies of Ca^2+^ release sites have revealed that RyR clusters exhibit edge-to-edge spacing of less than 100 nm [17, 18]. This suggests that neighboring sites may be functionally coupled through local Ca^2+^ diffusion. Therefore Ca^2+^ diffusion between SM compartments was implemented to reflect Ca^2+^ transport across steep [Ca^2+^] gradients on the periphery of the release site during release [19]. The SM compartment was modeled as a cylinder encircling the TT membrane with inner radius 100 nm, outer radius 180 nm, and 1 μm axis. It was assumed that 50% of NCX are located in the TT membrane of the SM compartment, and the remaining 50% are in the sarcolemmal membrane of the cytosolic compartment [20, 21]. Ca^2+^ in the SM is buffered by calmodulin and sarcolemmal binding sites. Ca^2+^ transport rates from the SM to the cytosol and between SM compartments were constrained to yield a realistic Ca^2+^ wave threshold (~100–150 μmol/[L cytosol]) [22] and velocity (50–100 μm/s) [4] in the baseline model.

Each spatial site, with location defined by coordinate (i,j,k), is represented by a set of ordinary differential equations describing local Ca^2+^ transport (Fig 1A). [Ca^2+^] is assumed to be uniform within the local JSR ([Ca^2+^]_JSR,i,j,k_), dyadic subspace ([Ca^2+^]_d,i,j,k_), and SM ([Ca^2+^]_SM,i,j,k_) compartments. Spatial Ca^2+^ diffusion is modeled as transport between SM compartments of adjacent release sites in the 3D lattice (Fig 1B). Model equations and parameters are given in S1 Equations and S1 Table.

Global compartments are used to represent average cytosolic [Ca^2+^]_i_ and network SR [Ca^2+^] ([Ca^2+^]_NSR_). The use of global compartments to represent these quantities eliminates two Ca^2+^ diffusion parameters and provides a three-fold reduction in the number of Ca^2+^ diffusion terms, thus substantially reducing model complexity and computational burden. While these simplifications result in over-estimation of the rise in [Ca^2+^]_i_ and fall of [Ca^2+^]_NSR_ at sites far away from an initiating Ca^2+^ wave, the model produces realistic spatiotemporal dynamics of spontaneous Ca^2+^ release and DADs.

Each release site contains a set of 48 RyRs and 8 LCCs that gate stochastically according to Markov chain models. The LCC model is as described in the Greenstein-Winslow model [13], with adjustments made to the rate of Ca^2+^-dependent inactivation (see S1 Text). Briefly, the LCC inactivation rate is a saturating function of [Ca^2+^]_SS_, which was necessary to reproduce inactivation kinetics consistent with the original model. Note, however, that as a result AP duration does not substantially decrease at reduced SR Ca^2+^ loads as exhibited previously [13]. RyR gating is described by a minimal two-state Markov model based on the work of Williams et al. [23], described in detail in Walker et al. [19]. Briefly, mean open time of each channel is 2 ms, and the opening rate is given by

$$r_{o}=\phi k^{+}{\left({\left[Ca^{2+}\right]}_{SS,i,j,k}\right)}^{\eta }$$ (1)

where k^+^ = 1.107 × 10^−4^ ms^-1^ μM^-η^ is the opening rate constant, η = 2.1 is the Ca^2+^ Hill coefficient, and *ϕ* is a [Ca]_JSR,i,j,k_-dependent regulation term given by

$$\phi =0.8025+{\left[\frac{{\left[Ca^{2+}\right]}_{JSR,i,j,k}}{1.5mM}\right]}^{4}$$ (2)

### Fiber model

The 3D cell model was incorporated into a tissue-scale model of a 1D fiber of myocytes. Fig 1C depicts the multiple biological scales represented in the model, from single ion channels to the multicellular fiber. The 3D cell model was augmented with a current carried by the gap junctions at either end of each cell (I_gap_). The current from cell i into an adjacent cell i+1 is given by:

$$I_{gap,i,i+1}=g_{gap}(V_{i}-V_{i+1})$$ (3)

where g_gap_ is the gap junction conductance, which was adjusted to yield a conduction velocity of 55 cm/s. The membrane potential in the fiber was solved using the Crank-Nicolson method [24] with 50 μs time steps. Operator splitting was used to explicitly solve each cell model using an embedded adaptive Runge-Kutta method described previously [13].

### Beta-adrenergic stimulation

Sympathetic stimulation of the heart occurs through β-adrenergic receptor activation, which activates intracellular signaling pathways, most notably Protein Kinase A (PKA) and Ca^2+^/calmodulin-activated protein kinase II (CaMKII), that increase contractility [25]. β-adrenergic stimulation is also known to be pro-arrhythmic, and can contribute to spontaneous Ca^2+^ release [26] in pathological conditions. Cell model parameters were modified to reflect the effects of acute β-adrenergic stimulation. LCC open probability was increased [27] by changing the fraction of gating LCCs from 25% to 60% and setting 3–5% of the channels to gate in a high-activity mode in which the mean open time was increased from 0.5 to 5.8 ms [28, 29]. Enhanced activation of inward currents was implemented for I_Kr_ using modifications described previously [29] and for I_Ks_ by shifting the voltage-dependence of activation by -35 mV and increasing conductance by 40% [30]. SR Ca^2+^ loading was facilitated by reducing SERCA pump K_d_ for [Ca^2+^]_i_ by 50% [31]. RyR opening rate was increased by either 50% to reflect increased SR Ca^2+^ leak observed in experimental studies [32] or 400% to reproduce pathological behavior after ouabain overdose [33]. Unless otherwise noted, these conditions were applied to all simulations in this study.

## Results

### Model properties

**Fig 2:**
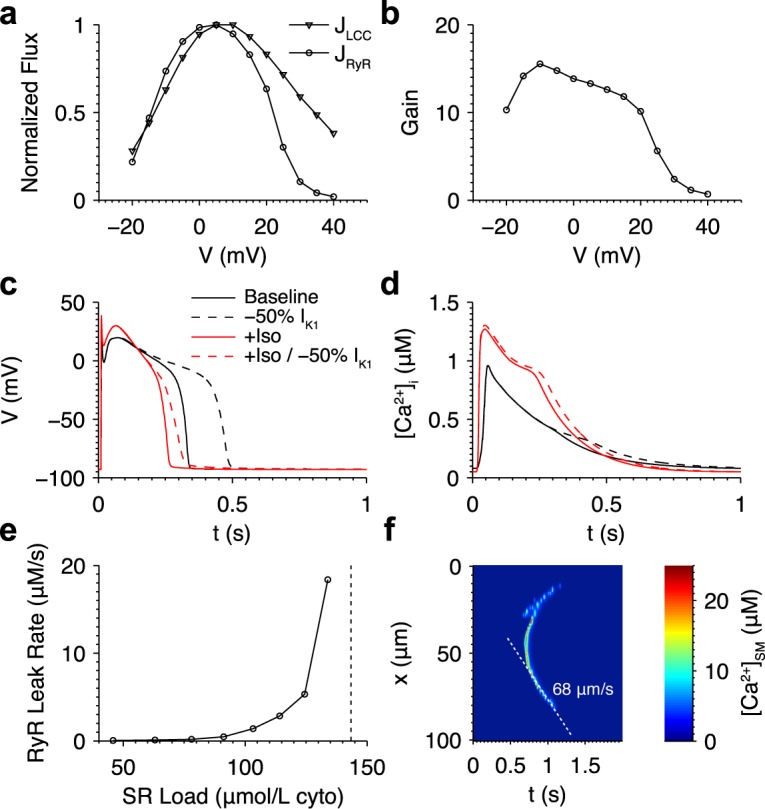
Ventricular myocyte model properties. (a) Normalized peak LCC (triangles) and RyR (circles) flux when the membrane was stepped to test potentials between -20 and 40 mV. (b) Excitation-contraction coupling gain, defined as the ratio of the normalized peak RyR to LCC Ca^2+^ flux at each test potential. (c) Representative action potentials and (d) [Ca^2+^]_i_ transients under control conditions (solid black), with 50% I_K1_ inhibition (dashed black), with β-adrenergic stimulation (solid red) conditions, and with β-adrenergic stimulation and 50% I_K1_ inhibition (dashed red). (e) Cell-wide SR Ca^2+^ leak rate via RyRs at varying SR Ca^2+^ loads in the baseline model. The dotted line indicates the lowest SR Ca^2+^ load tested that exhibited spontaneous Ca^2+^ waves. (f) Example linescan plot showing a Ca^2+^ wave under β-adrenergic stimulation.

In order to reproduce protocols designed to measure ECC properties, membrane potential was stepped to varying test potentials for 200 ms from a holding potential of -80 mV. The dependence of normalized peak RyR and LCC Ca^2+^ fluxes on the test potential and corresponding ECC gain values are similar to those observed experimentally [34] (Fig 2A and 2B). The peak normalized RyR flux curve is right-shifted with respect to that of the LCC flux curve. The ECC gain is 13.8 at 0 mV and decreases monotonically with increasingly depolarized test potentials. The lack of high gain at potentials below -10 mV is a result of consolidating the four distinct dyadic subspace compartments utilized in the previous Greenstein-Winslow canine ventricular myocyte model [13] into one, a simplification of the Ca^2+^ release site model resulting in improved computational efficiency, but which attenuates the local [Ca^2+^]_d_ signal caused by the brief high-amplitude unitary LCC currents characteristic in this potential range.

Under control conditions, model APs and [Ca^2+^]_i_ transients are similar to those of normal canine ventricular myocytes [35], with an AP duration (APD), defined as the time to reach 90% repolarization, of approximately 320 ms (Fig 2C and 2D). The model reaches a stable steady state after ~ 10 seconds when paced at 1 Hz (S2 Fig). The NCX current during an action potential (S1 Fig) is in agreement with experimental and theoretical studies [36–38]. Reducing I_K1_ density by 50% prolongs the action potential to 469 ms due to the reduction in repolarizing current. Note that this is shown for the first paced beat with identical initial conditions as the baseline model, as the model fails to repolarize under these conditions after continued pacing (see Discussion). Simulating the effect of β-adrenergic stimulation increases the amplitude of the AP plateau as well as [Ca^2+^]_i_ transient amplitude and decay rate in addition to decreasing the APD to ~255 ms [29]. Additionally reducing I_K1_ density by 50% resulted in a marked increase in APD to ~300 ms at steady state. This prolonged AP increased Ca^2+^ loading, resulting in marginally higher systolic [Ca^2+^]_i_.

[Ca^2+^]_NSR_ was clamped at increasing values to test the relationship between SR Ca^2+^ load and leak. The model exhibits an exponential leak-load relationship that is similar to experimental estimates [39, 40] (Fig 2E). Spontaneous Ca^2+^ waves form at a threshold SR Ca^2+^ load, at which wave fronts of propagating Ca^2+^ sparks emanate from random regions of high spark activity. Fig 2F shows a plot of SR Ca^2+^ leak along a cross-section through the center of the cell during a representative Ca^2+^ wave under β-adrenergic stimulation, this wave occurred at a lower SR Ca^2+^ load (82 μmol/L cytosol) compared to under baseline conditions due to greater RyR Ca^2+^ sensitivity. The wave shape and velocity of 68 μm/s are similar to those observed in experimental studies [4].

### Delayed afterdepolarizations during pacing

Liu et al. demonstrated that ouabain overdose causes accumulation of [Na^+^]_i_, leading to Ca^2+^ overload and DADs [33]. In addition, the authors showed that the production of reactive oxygen species, which are known to oxidize RyRs [41] and CaMKII [42], both of which enhance RyR activity, contributed to DAD generation. To induce Ca^2+^ overload, model parameters were modified to simulate β-adrenergic stimulation and to reflect the conditions in Liu et al. [33] by inhibiting the Na^+^/K^+^ ATPase by 90%, which leads to accumulation of intracellular Na^+^ (>20 mM).

**Fig 3:**
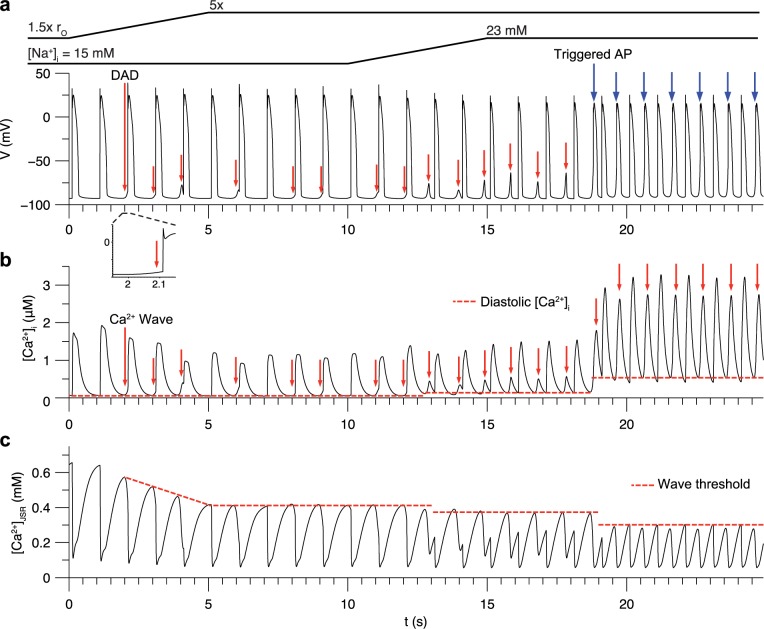
DADs induced by Ca^2+^ overload during 1 Hz pacing under β-adrenergic stimulation in the myocyte model. (a) Sub-threshold DADs (red arrows) and triggered APs (blue arrows) in between paced APs. Inset illustrates how the first DAD occurred just prior to the next stimulus. (b) Spontaneous [Ca^2+^]_i_ transients caused by underlying Ca^2+^ waves. Dotted lines indicate diastolic [Ca^2+^]_i_. (c) Average [Ca^2+^]_JSR_. Dotted lines indicate threshold for Ca^2+^ overload that resulted in spontaneous Ca^2+^ waves. This threshold decreased over the course of the protocol due to increased RyR sensitivity, increased [Na^+^]_i_, and elevated diastolic [Ca^2+^]_i_.

To demonstrate the emergence of DADs in the model, we simulated a protocol in which the RyR opening rate and [Na^+^]_i_ were controlled over time. β-adrenergic stimulation was applied and the cell was paced at 1 Hz. First, [Na^+^]_i_ was fixed to 15 mM and the RyR opening rate was ramped from 1.5x to 5x that of baseline over t = 0 to 5 s. Intermittent Ca^2+^ waves, triggered by overload of JSR Ca^2+^, cause spontaneous [Ca^2+^]_i_ transients, which began to occur (Fig 3A). RyR sensitization initially causes an increase in [Ca^2+^]_i_ transient peak from 1.73 to 1.93 μM, followed by a decrease (Fig 3B) as the Ca^2+^ wave threshold decreases (Fig 3C) and cellular Ca^2+^ is extruded by NCX. Many of these events begin just prior to (<100 ms before) the next stimulus (Fig 3A inset). Prominent DADs occur at t = 4 and 6 s. This causes lower Ca^2+^ transient amplitude in the following beats due to the lost Ca^2+^ stores. The threshold SR load for Ca^2+^ waves was reduced to 80 μmol/[L cytosol] compared to the baseline model threshold of 140 μmol/[L cytosol] (see Fig 2E). This is due to high [Na^+^]_i_ and the greater RyR opening rate, which increases Ca^2+^ spark frequency and results in Ca^2+^ wave nucleation and propagation.

To induce triggered APs, [Na^+^]_i_ was ramped from 15 to 23 mM over t = 10 to 15 s. This causes Ca^2+^ waves to form more readily by reducing extrusion of Ca^2+^ via NCX in the SM compartment, thus elevating [Ca^2+^]_d_ at sites adjacent to Ca^2+^ sparks and increasing the probability of Ca^2+^ spark propagation. Note that elevated diastolic [Ca^2+^]_i_ has been implicated in DAD formation in experimental studies [8, 43]. Diastolic [Ca^2+^]_i_ was at first ~140 nM when the DADs are sub-threshold. Higher [Na^+^]_i_ decreases the delay until the DADs, resulting in higher diastolic [Ca^2+^]_i_. A DAD of sufficient amplitude to activate I_Na_ occurs at t = 18.7 s and triggers a spontaneous AP. Triggered APs result in greater spontaneous [Ca^2+^]_i_ transients due to activation of LCCs, and the cells exhibit elevated diastolic [Ca^2+^]_i_ in the range of ~470–520 nM. This causes a reduction in the SR Ca^2+^ load threshold for spontaneous release to 63 μmol/[L cytosol] due to the resulting increase in RyR opening rate and Ca^2+^ spark frequency. Note that under these conditions, triggered APs exhibit pacemaker-like automaticity, occurring every ~570 ms after cessation of pacing (S3 Fig) but stop when [Na^+^]_i_ is lowered below ~20 mM. This behavior is consistent with spontaneous contractions observed in mouse heart in the presence of ouabain [44].

The protocol also produced changes in AP duration (APD) (S3 Fig). The first beat has an APD of 235 ms, 27% shorter than under normal conditions due reduced inward NCX current in the presence of elevated [Na^+^]_i_, [45]. Upon RyR sensitization, APD decreases to ~215 ms due to decreased inward NCX current accompanying the smaller [Ca^2+^]_i_ transients. Further increasing [Na^+^]_i_ to 23 mM reduced APD to 150 ms. A previous study showed that APD increases following spontaneous Ca^2+^ release due to slowed Ca^2+^-dependent inactivation of the LCCs [46]. The model does not reproduce this behavior because of the LCC Markov chain has a saturating dependence on [Ca^2+^]_d_ and is therefore not sensitive to Ca^2+^ load (see Discussion). Rather, the lower [Ca^2+^]_i_ transient results in less inward NCX current, thus reducing time to repolarization.

These results demonstrate how the model reproduces SR Ca^2+^ overload as a driver of DADs under pathophysiological conditions. Elevated RyR sensitivity and [Na^+^]_i_ accumulation led to DADs of sufficient amplitude to trigger action potentials during the diastolic intervals. In addition, these results illustrate the interplay between [Ca^2+^]_i_ and SR load dynamics during spontaneous Ca^2+^ release.

### Effect of SR Ca^2+^ load on DADs

**Fig 4:**
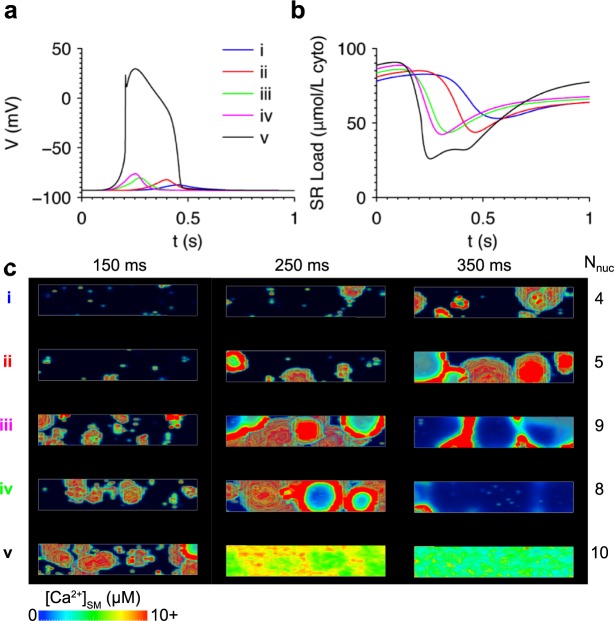
Elevating SR Ca^2+^ load accelerated Ca^2+^ wave formation and increased DAD amplitude. (a) DADs resulting from initializing SR Ca^2+^ load to five different values as shown in (b). (c) Volume renderings of Ca^2+^ in the simulations at three time points illustrating the greater spontaneous Ca^2+^ wave activity at higher SR Ca^2+^ loads. The number of Ca^2+^ wave nucleation sites (N_nuc_) is tabulated for each simulation. All simulations initialized with [Na^+^]_i_ = 10 mM.

The relationship between SR Ca^2+^ load and spontaneous Ca^2+^ release was investigated. Simulations were run using initial conditions reflecting the cell state just prior to the moment when SR Ca^2+^ load reaches the Ca^2+^ wave threshold following an AP. Initial [Ca^2+^]_i_ was set to 150 nM, similar to the level during the late decay phase of a cytosolic Ca^2+^ transient. Fig 4A shows DADs occurring at the five different values of initial SR Ca^2+^ load shown in Fig 4B. At the highest SR Ca^2+^ load, DAD amplitude is large enough to trigger an AP, as shown in simulation (v) in Fig 4A. Elevating SR Ca^2+^ load reduces the delay until the spontaneous release event, consistent with the observations of Wasserstrom et al. [10]. The increase in DAD amplitude is consistent with a study by Schlotthauer and Bers, who demonstrated increased amplitude of caffeine-induced DADs at higher SR Ca^2+^ loads [47].

Fig 4C shows volume renderings of [Ca^2+^]_SM_ at three time points in each simulation. The number of Ca^2+^ wave nucleation sites (N_nuc_) was defined as the number of independently formed wave front epicenters and was estimated by inspection of the volume renderings (S1 Movie). N_nuc_ generally increases with SR Ca^2+^ load, in agreement with experimental studies in intact heart [9, 10]. Therefore, the increase in SR Ca^2+^ load also increases RyR Ca^2+^ release flux (J_RyR_) by enhancing the synchrony of RyR opening and number of nucleation sites.

### Ensemble properties of DADs

**Fig 5:**
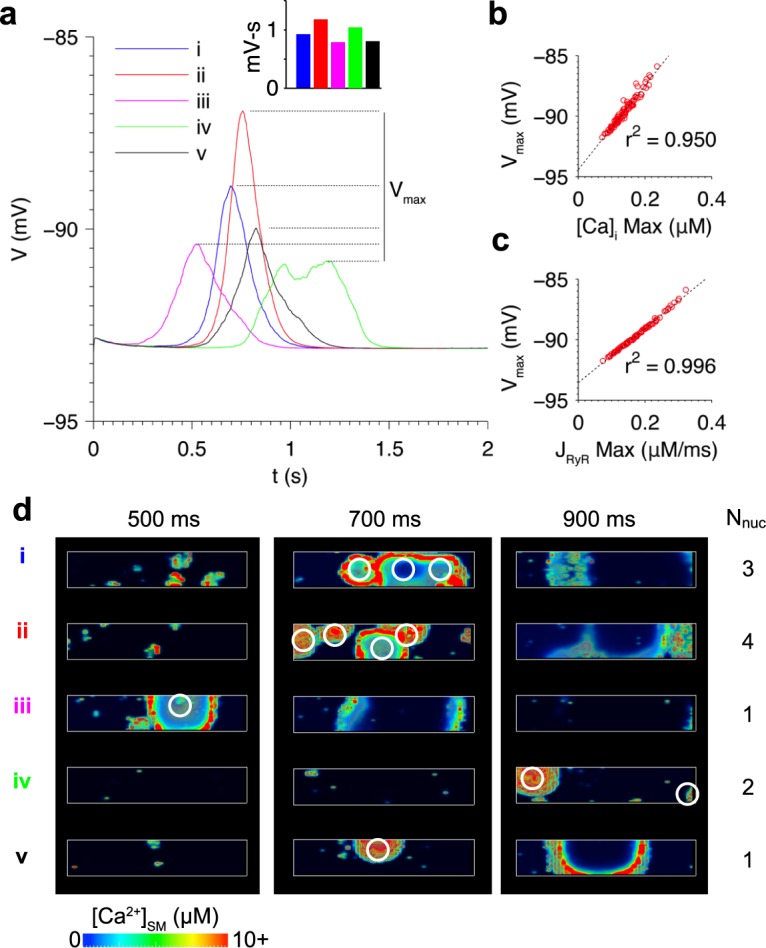
Variability of DAD timing and amplitude in five independent model realizations with identical initial conditions. (a) Variability in DAD timing and amplitude. Inset shows the area under the curve above resting potential of each DAD. Strong correlations were observed between V_max_ and the maxima of both the spontaneous [Ca^2+^]_i_ transient (b) and the RyR Ca^2+^ release flux (c). (d) Volume renderings of Ca^2+^ wave dynamics at three time points in each simulation. Approximate locations of the nucleation sites are circled in white. N_nuc_ was higher in simulations *i* and *ii* which had greater DAD amplitude. All simulations initialized with [Na^+^]_i_ = 10 mM.

We hypothesized that stochastic gating of the RyRs drives variability in Ca^2+^ wave dynamics and thus DAD amplitude and timing. To test this, five independent realizations were generated, each of which had identical initial conditions similar to simulation (i) from Fig 4. The pseudorandom number generator seed was varied among the realizations in order to produce independent patterns of RyR gating. Fig 5A shows the resulting DADs, which exhibit marked variability in timing and amplitude. Time of occurrence of the DAD peak varies from 520 to 1209 ms and peak amplitudes range from 2.3 to 6.2 mV. These DADs appear qualitatively similar to experimental observations in rat myocytes, with delays ranging by ~1 s and amplitude ~2-fold, although the SR Ca^2+^ load was not reported [6]. The area under the curve of each DAD, measured relative to the resting potential, varied from 0.79 in (iii) to 1.18 in (ii) (50% greater). This roughly correlated with DAD amplitude, with the exception of the prolonged DAD (iv), which had the second greatest area under the curve despite having the lowest amplitude. Thus substantial DAD variability can be attributed to the stochastic nature of RyR gating.

Spontaneous Ca^2+^ release generates DADs by driving an inward current through NCX [6]. Imaging, electrophysiological, and modeling [38] studies have all suggested that NCX senses a [Ca^2+^]_SM_ that is greater than [Ca^2+^]_i_ because it can be localized near the release sites [16, 20, 21]. Therefore, the driving force for inward NCX current is likely to be determined, in part, by the aggregate number of concurrent Ca^2+^ sparks occurring across the cell. This is consistent with a study showing that peak Ca^2+^ release flux is strongly correlated with the likelihood of ectopic activity [48]. An ensemble of 98 independent simulations were performed to determine how peak [Ca^2+^]_i_ and J_RyR_ are related to DAD amplitude. Fig 5B and 5C show there is a strong linear correlation between the peak membrane potential during the DAD (V_max_) and the maximum of [Ca^2+^]_i_ during the spontaneous [Ca^2+^]_i_ transient (r^2^ = 0.950). There is an even stronger relationship between V_max_ and the peak J_RyR_ value (r^2^ = 0.998). This confirms that the inward NCX current is primarily driven by J_RyR_ via the resulting rise in [Ca^2+^]_SM_.

Note that J_RyR_ reflects the combined Ca^2+^ release flux associated with all Ca^2+^ sparks occurring at any time. It was therefore expected that the variability in DADs was the result of spatio-temporal variations in Ca^2+^ wave dynamics. Fig 5D and S2 Movie show volume renderings of the Ca^2+^ waves in each simulation. Waves emanate from nucleation sites of high Ca^2+^ spark activity. For example, in the simulation (iv) of Fig 5D at 700 ms, a large cluster of Ca^2+^ sparks is visible at the left end of the cell, and by 900 ms a wave front of Ca^2+^ is seen propagating radially away from this cluster site. The random nature of nucleation site locations results in DAD variability across simulations. Simulations *i* and *ii* are both associated with the highest-amplitude DADs as well as the greatest number of nucleation sites, as shown in Fig 5D. These nucleation sites are spaced widely across the cell, resulting in independent propagating wave fronts of high [Ca^2+^]_SM_ that drive inward I_NCX_. The remaining three simulations have lower amplitudes due to fewer nucleation sites. Note that in simulation *iv*, two separate Ca^2+^ waves form over 100 ms apart, resulting in a prolonged low-amplitude DAD with two peaks. These results are consistent with the strong correlation between V_max_ and maximum J_RyR_, which reflects the timing and pattern of Ca^2+^ wave formation.

### Dependence of DAD distribution on Ca^2+^ load and I_K1_ density

**Fig 6:**
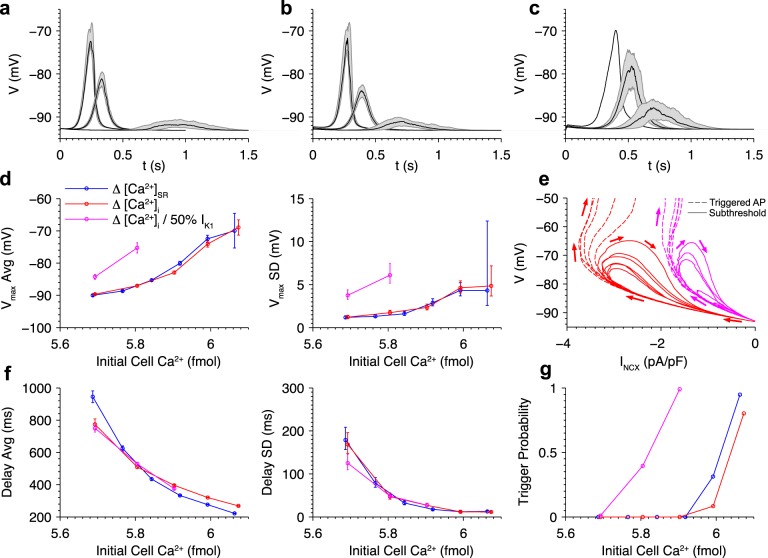
Roles of cytosolic and SR Ca^2+^ and I_K1_ density in DAD distribution. DAD distributions when initial (a) [Ca^2+^]_SR_ and (b) [Ca^2+^]_i_ were varied in the presence of β-adrenergic stimulation. (c) DAD distributions when varying initial [Ca^2+^]_i_ after reducing I_K1_ density by 50%. Shaded regions indicate the middle quartiles of the membrane potential for sub-threshold events out of 96 simulations. Dark lines indicate median values. Statistics of V_max_ (d) of sub-threshold events, phase plane plot of V and NCX current (e), and statistics of delay until DAD peak (f) are shown when SR [Ca^2+^] was varied (blue), [Ca^2+^]_i_ was varied (red), and [Ca^2+^]_i_ was varied with 50% reduction in I_K1_ density (magenta) as a function of initial total cell Ca^2+^. Error bars indicate standard deviation (SD) of the estimates. Panel (e) shows representative sub- and supra-threshold DADs for the baseline model and I_K1_ reduced by 50% with [Ca^2+^]_i_ initially at 3.5 and 2 μM, respectively. Arrows indicate direction of time. (g) Dependence of triggered AP probability on initial total cell Ca^2+^. All simulations initialized with [Na^+^]_i_ = 10 mM.

In this section, the statistical relationships between Ca^2+^ loading and DAD amplitude and timing in ensemble simulations are investigated. Fig 6A shows variability in sub-threshold DAD properties when initial SR Ca^2+^ load is varied. The dark lines indicate the median value of membrane voltage over time, and the shaded regions illustrate the range of the second and third quartiles (25^th^ to 75^th^ percentile). Consistent with findings from the individual cell simulations of Fig 4, DAD delay decreases and amplitude increases with increasing SR Ca^2+^ load. The peaks of the upper and lower bounds are -92.4 and -90.6, -82.7 and -79.5, and -73.2 and -68.1 mV for initial SR [Ca^2+^] values of 0.32, 0.36, and 0.40 mM, respectively. This suggests that there is an increase in DAD amplitude variability at higher SR Ca^2+^ loads. Note also that the width of the shaded regions decreases as SR load increased, reflecting increased DAD synchrony.

Recall that diastolic [Ca^2+^]_i_ plays a critical role in determining the SR Ca^2+^ wave threshold during pacing (Fig 3). The effect of increasing [Ca^2+^]_i_ on the DAD distribution with SR Ca^2+^ load held constant was next tested (Fig 6B). Elevating [Ca^2+^]_i_ yields identical effects as seen when increasing SR Ca^2+^ load, with both reducing the delay and increasing the amplitude of DADs.

The inward rectifier K^+^ current, I_K1_, is the primary membrane current that stabilizes V at the cell’s resting potential and plays a critical role in protecting the cell from triggered APs. Down-regulation of I_K1_ is associated with ventricular arrhythmias in diseases such as heart failure [49], Andersen’s syndrome [50], and long QT syndrome [51]. Fig 6C shows DAD distributions when I_K1_ density is reduced by 50% and [Ca^2+^]_i_ is varied. These changes increase DAD amplitude and variability compared to cells with normal I_K1_. Note that in the 350 nM [Ca^2+^]_i_ case, only one of the 98 realizations produces a sub-threshold DAD, while all others exhibit triggered APs.

Fig 6D shows the average and SD of V_max_ as a function of total cell Ca^2+^, defined as the total of buffered and free Ca^2+^ in the cytosol and SR. Changes in either initial [Ca^2+^]_i_ or [Ca^2+^]_SR_ (which implicitly include changes in Ca^2+^-bound buffer concentrations to their equilibrium values) are reflected in initial total cell Ca^2+^. The effect of 50% I_K1_ density reduction on the [Ca^2+^]_i_-dependence of V_max_ was also analyzed. These results demonstrate two important conclusions.

First, in the baseline model with normal I_K1_, the distributions of V_max_ as a function of initial total cell Ca^2+^ are identical in both cases, as shown by overlap of the blue (initial [Ca^2+^]_i_ varied) and red (initial [Ca^2+^]_SR_ varied) traces. This strongly suggests that DADs are driven by both [Ca^2+^]_i_ and [Ca^2+^]_SR_ and is consistent with the notion that total cell Ca^2+^ plays a major role in setting the DAD distribution, as observed in other models [52].

Second, reducing I_K1_ by 50% causes a marked increase in the average and SD of V_max_. In this case, V_max_ was more sensitive to inward I_NCX_ during the DAD because I_K1_ rectification reduces its outward current upon membrane depolarization which increases the contribution of I_NCX_ to the net membrane current, and this imbalance between the currents becomes greater with reduction in I_K1_ density. This phenomenon is illustrated in a phase plane plot of example simulations at baseline and 50% I_K1_ densities exhibiting both sub- and supra-threshold DADs (Fig 6E). For a given NCX current, V rises substantially higher at 50% I_K1_ density compared to the baseline model. The rightward shift of the trajectories also illustrates the ~50% reduction in peak NCX current required to trigger an AP (~3.4 to ~1.7 pA/pF). Therefore, DAD amplitudes are greater for a given I_NCX_ amplitude, causing an increase in the mean. Similarly, amplitude SD is also increased. At the lowest Ca^2+^ load tested, 50% IK1 reduction caused ~3x greater SD, which is mainly accounted for by the fact that the amplitude is on average ~2.6x greater.

DAD delay and synchrony is also strongly correlated with total cell Ca^2+^, as measured by the distribution of the time until the DAD peak occurred (Fig 6F). Increasing initial total cell Ca^2+^ reduces DAD delay and increases synchrony, similar to the observations of Wasserstrom et al. [10]. In the baseline model, the standard deviation of the delay was 178 ms at the lowest SR load tested. This variability is similar to that measured by Wasserstrom et al. (162 ms) in whole rat heart paced at 1 Hz and elevated [Ca]_o_ = 7.0 mM. DAD delay does not considerably change with 50% I_K1_ reduction because it is determined primarily by the timing of Ca^2+^ wave formation, which is not affected by I_K1_ density.

With increasing initial total cell Ca^2+^, in the form of increased initial [Ca^2+^]_i_ or [Ca^2+^]_SR_, the higher DAD amplitudes makes it more likely that the threshold membrane potential for triggering an AP (~ -55 mV) will be reached. The probability that a DAD-triggered beat occurred is shown in Fig 6G. Increasing cell Ca^2+^ results in a steep increase in trigger probability. This switch-like behavior is due to the DAD amplitude reaching threshold AP-triggering potential with high certainty. Reducing I_K1_ by 50% reduces the critical Ca^2+^ load at which APs are triggered, consistent with the observed increase in DAD amplitude. The effect is significant: at normal I_K1_ density zero of 96 cells exhibit triggered APs when initial [Ca^2+^] is 5.8 fmol, but reducing I_K1_ density by 50% increases the probability to ~ 0.5. Thus I_K1_ density plays a critical role in modulating the Ca^2+^ load at which arrhythmia-triggering events occur.

### Probabilistic triggered activity in a paced fiber of myocytes

**Fig 7:**
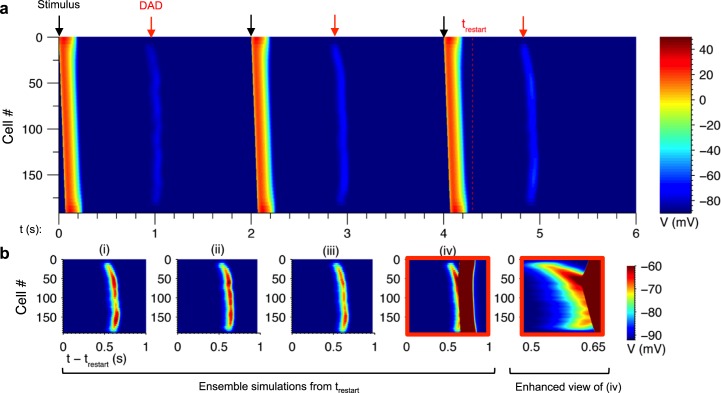
Probabilistic triggered activity caused by DADs in the fiber model. (a) Fiber of 192 cells paced at 0.5 Hz by applying a stimulus current for 2 ms to the first two cells in the fiber (black arrows). DADs occurred between paced beats (red arrows). (b) Ensemble simulations (i)-(iv) initialized to the state at time t_restart_ in panel (a). Realization (i) corresponds to the simulation in panel (a). Enhanced view of (iv) is shown at right.

In the previous section, it was shown that triggered APs occur with a probability that depends on Ca^2+^ load and that varying I_K1_ density shifts the Ca^2+^ load dependence. Simulations were performed to test whether the model could produce probabilistic triggered activity in a 1D fiber of myocytes during pacing, where electrotonic loading of any cell by adjacent cells becomes important. Fig 7A shows the membrane potential of a fiber paced at 0.5 Hz under conditions similar to those in Fig 3 with elevated [Na^+^]_i_ set to 19 mM, as may be observed in heart failure [53]. Note also that [Na^+^] sensed by membrane channels during contraction may be higher than [Na^+^]_i_ in the bulk cytosol due to local buildup of Na^+^ imported by NCX [54]. To reflect a state of pathological remodeling, I_K1_ density and gap junction conductance were each reduced by 50% as observed in HF [49, 55]. To limit boundary effects, [Na^+^]_i_ was set to 10 mM in the outer twenty-four cells near each end of the cable in order to prevent DAD generation. DADs, appearing as faint bands of depolarization between the paced beats, and reach V_max_ values between approximately -70 and -60 mV. This is consistent with experimental observations of synchronized spontaneous Ca^2+^ release causing DADs in intact heart following rapid pacing [9].

The model exhibited considerable variability in V_max_ along the length of the fiber. To investigate the source of this variability, the state of the model at time t_restart_ immediately after the third paced beat was recorded. A set of three independent realizations were run, each starting from this same model initial condition but with simulations performed using different initial pseudorandom number generator seeds. These realizations therefore differ in the particular pattern of LCC and RyR channel gating.

Fig 7B shows: (i) the DAD from the original simulation in Fig 7A at 4.9 s; and (ii)-(iv) the three additional simulations initialized at t_restart_. Each simulation exhibits substantial differences in DAD amplitude along the fiber. In panel (iv), a spontaneous traveling action potential wave is observed. The wave originates from a region of locally high DAD amplitude near cell #50 and emanates in either direction along the fiber.

These results illustrate that arrhythmic events can occur probabilistically in tissue. By restarting the simulations immediately prior to the DADs, it became evident that the variability in DAD amplitude was due to variations in the stochastic events that occurred in this brief time window. Taken together with the results from Figs 5 and 6, the variability in DAD amplitude in the fiber can therefore be attributed to the underlying randomness of Ca^2+^ wave dynamics and thus stochastic RyR gating.

### Roles of Ca^2+^ loading, I_K1_ density, and g_gap_ in fiber DADs

**Fig 8:**
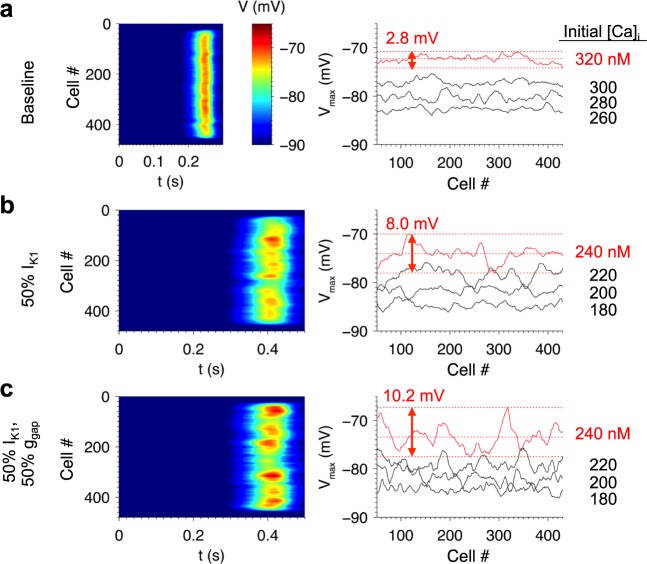
Roles of initial [Ca^2+^]_i_, I_K1_ density, and g_gap_ in DAD variability in the fiber model. (a) Spatiotemporal profile of V (left) and V_max_ profile (right) in a 480-cell fiber under baseline conditions. Similar simulations are shown with 50% I_K1_ reduction (b) and both 50% I_K1_ and 50% g_gap_ (c). The range of V_max_ values are indicated for the red traces, which correspond to the images on the left. To avoid boundary effects, the outer 24 cells on either end were initialized to normal SR loads. The inner cells were initialized to identical initial conditions to those of Fig 6. All simulations initialized with [Na^+^]_i_ = 10 mM.

Conditions reflecting pathological remodeling influenced the distribution of V_max_ in the fiber. Fig 8A shows fiber simulations when all cells are initialized to identical initial conditions with Ca^2+^ overload and β-adrenergic stimulation. The resulting DADs resemble those from the paced fiber shown in Fig 7. As in individual cells, V_max_ increases with initial [Ca^2+^]_i_. At the highest [Ca^2+^]_i_, V_max_ lies between -74.1 mV and -70.7 mV, a range of 3.4 mV. There is notably less variability than in individual cells at a similar Ca^2+^ load (SD of 0.7 compared to 4.6 mV, see Fig 6D). This reduction in variability is due to electrotonic coupling through inter-cellular gap junctional conductance, g_gap_, which attenuates spatial gradients in V.

Fig 8B shows similar simulations where I_K1_ density was reduced by 50%. This results in greater fluctuations of V_max_ over a range of 8.0 mV (SD 1.5 mV) compared to baseline. This is consistent with the increase in V_max_ SD from 1.7 to 6.1 mV when I_K1_ density was decreased to 50% in isolated cells (see Fig 6D) and also reflects a substantial reduction in V_max_ variability due to electrotonic coupling in the fiber. Reducing g_gap_ by 50% in addition to the 50% I_K1_ reduction further amplifies V_max_ spatial fluctuations, resulting in a range of 10.2 mV (SD 2.2 mV, Fig 8C). The increase in variability arises from the reduced electrotonic load experienced by each cell. These results demonstrate how perturbations of I_K1_ and g_gap_ increase the likelihood of observing large DADs.

### Overview of method for estimating rare event probabilities

The DAD results presented thus far indicate that spatial fluctuations in V_max_ can result in a triggered beat emanating from a cluster of cells in which V_max_ exceeds the threshold voltage (~ -55 mV). For a fiber of a given length, the probability of this event depends upon both the mean V_max_ and the likelihood of a deviation from the mean with sufficient amplitude to reach threshold. Under conditions where the mean V_max_ is far below threshold, however, it is unclear whether it would be plausible to observe a sufficiently large fluctuation that causes a triggered beat. We sought to characterize the likelihood of such events by estimating the upper tail of the V_max_ distribution.

In Fig 5, we showed that V_max_ was strongly correlated with J_max_ in DAD simulations of isolated cells. In tissue, however, electrotonic coupling attenuates DAD amplitude by drawing current to neighboring cells via gap junctions. However, if spontaneous Ca^2+^ release occurs synchronously in a cluster of cells, the potential gradient between cells is smaller, thus reducing the gap junction current and increasing DAD amplitude. Therefore the V_max_ of a given myocyte in tissue depends on the relative timing and amplitude of Ca^2+^ release in nearby cells.

We hypothesize that extreme deviations in V_max_ are caused by rare spatially clustered synchronized Ca^2+^ release events. However, estimating the probability of extremely rare events (e.g. 1 in 10^6^) by direct simulation is computationally prohibitive using the full biophysical model since it would require potentially millions of simulations. Therefore, a method was developed for estimating the probability of such events using the output from a limited set of simulations.

As shown in the previous sections, stochastic RyR activity gives rise to random Ca^2+^ wave patterns and thus variable profiles of J_RyR_. Therefore J_RyR_ is a stochastic process that is dependent on complex microscopic events, which are computationally intensive to sample. The method leverages the fact that J_RyR_ has a relatively simple distribution by resampling J_RyR_ profiles from a set of simulations that is sufficiently large to approximate the distribution of J_RyR_. Multiple realizations of release events (i.e., the J_RyR_ values from each separate simulation of the full cable model) are generated using a resampling method in which cell positions along the cable are shuffled to produce independent, distinct realizations. The key to this approach is the fact that spontaneous Ca^2+^ release events are decoupled across the cells in the fiber (see Discussion).

In principle, in using this approach one could develop a modified tissue model in which J_RyR_ in each cell is fixed to one of the resampled J_RyR_ profiles rather than being simulated, thus greatly reducing the computational burden. However, this would still require immense computational power to simulate the remaining differential equations when estimating the probability of extreme events. The second part of the method further increases computational efficiency by fitting a linear spatial-averaging model to predict V_max._from J_RyR_ alone. This permits the rapid estimation of the greatest V_max_ in a 1D tissue model in millions of simulations and thus can estimate the probability that the cable will reach a proarrhythmic threshold potential.

The following sections describe this method and validate its accuracy for estimating DAD probabilities. We then apply this new technique to show how myocyte properties within a fiber, using I_K1_ density and g_gap_ as examples, affect the likelihood of rare large amplitude DADs.

### Filtering method for estimating V_max_ from J_RyR_

**Fig 9:**
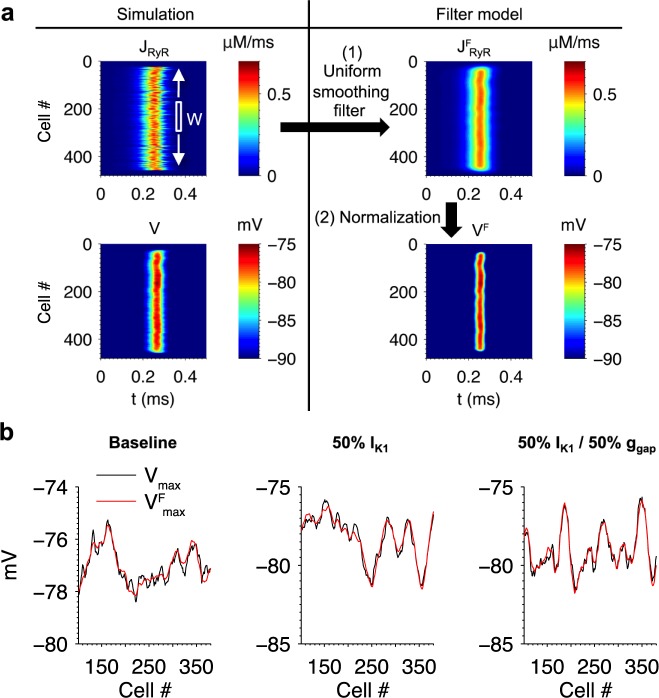
Filtering method for estimating V_max_ from the spatiotemporal J_RyR_ profile in a fiber. (a) Illustration of the filtering method for an example fiber simulation. A uniformly-weighted spatial smoothing filter of width W was applied to the spatiotemporal J_RyR_ profile. The maximum values of the filtered profile were then normalized to obtain an estimate of the voltage profile (see Eq 6). (b) The filtering method was applied to three fiber simulations using the conditions indicated above each plot. The filtering method estimate (red) accurately reproduced V_max_ from the original simulation (black). Parameters are listed in Table 1.

A filtering method was developed for estimating V_max_ from the spatiotemporal profile of J_RyR_ in a single 1D fiber simulation. The left column of Fig 9A shows the simulation from Fig 8A where [Ca^2+^]_i_ was initialized to 300 nM, and the right column illustrates the steps used in the filtering method. The first step in the method is to apply a uniform averaging filter to J_RyR_ at each point in time to obtain a spatially smoothed profile

$$J_{RyR}^{f}(x,t)=\frac{1}{W}\sum_{k=-(W-1)/2}^{(W-1)/2}J_{RyR}(x+k,t)$$ (4)

where x refers to cell index, t refers to time, and W is the width of the filter (an odd integer). The rational for doing this is that membrane potential fluctuations of the cell at position x is influenced not only by the way its own complement of NCX responding to the local J_RyR_, but is also influenced by membrane potential fluctuations produced in response to J_RyR_ and NCX activity in neighboring cells. The filter width W will therefore depend on the strength of gap junction coupling. For each cell, the maximum J^f^_RyR_ value over all time was computed as

$$J_{\max}^{f}(x)=\underset{t}{\max}\left\{J_{RyR}^{f}(x,t)\right\}$$ (5)

The value of W maximizing the correlation coefficient between V_max_ and J^f^_max_ was calculated at each different value of gap junction coupling conductance used. J^f^_max_ was then normalized to obtain estimates of V_max_ using the formula

$$V_{\max}^{f}(x)=\mu_{V}+\frac{\sigma_{V}}{\sigma_{V}}\left(J_{\max}^{f}(x)-\mu_{J}\right)$$ 6

where μ_V_ and σ_V_ are estimates of the mean and SD respectively of V_max_, and μ_J_ and σ_J_ are estimates of the mean and SD respectively of J^f^_max_. These estimates are calculated from a single full 1D tissue simulation containing ~ 450 cells. Note how in the example of Fig 9A the estimated voltage profile V^f^ closely resembles that of V. This approach therefore leverages the strong linear correlation between J_RyR_ and V_max_ (Fig 5B and 5C) and the statistical independence of Ca^2+^ release events to dramatically reduce the number of simulations needed to estimate the probability distribution of membrane potential fluctuations.

The filtering method was applied to simulations of DADs with baseline conditions, with 50% I_K1_, and with both 50% I_K1_ and 50% g_gap_ (Fig 9B). In the non-baseline conditions, initial [Ca^2+^]_i_ was adjusted from 300 to 220 nM so that μ_V_ would be approximately equal to that of the baseline. The width W of the smoothing filter is dependent on the fiber model parameters and therefore was optimized separately for each case. Table 1 shows the parameter fits for each condition. The resulting fits of V^f^_max_ to V_max_ have high correlation coefficient values (r^2^ ≥ 0.90). All values of μ_V_ fall within a narrow range from -77.0 mV to -79.1 mV, while the values of μ_J_ in the two non-baseline conditions are less than half of the baseline value due to their lower Ca^2+^ loads.

**Table 1:** Parameters used for filtering method in Fig 9B.

	[Ca^2+^]_i_ (nM)	r^2^	μ_V_ (mV)	μ_J_ (μM ms^-1^)	σ_V_ (mV)	σ_J_ (μM ms^-1^)	W (cells)	S_V_
Baseline	300	0.900	-77.0	0.526	0.665	0.00873	43	1.77
50% I_K1_	220	0.946	-78.3	0.248	1.38	0.0124	49	2.27
50% I_K1_, 50% g_gap_	220	0.953	-79.1	0.242	1.34	0.0150	27	3.31

The increase in V_max_ variability in the two pathological conditions is reflected in the parameters of the filtering model. It can be shown that the quantity S_V_ = (σ_V_/σ_J_)/W scales with the SD of V^f^_max_ (see S1 Text). Recall that 50% I_K1_ reduction increased variability of V in the cell model (Fig 6). For this condition in the fiber, S_V_ is 28% larger compared to baseline, primarily reflecting the higher value of σ_V_. Imposing 50% g_gap_ results in a filter width of only 27 cells compared to 43 and 49 in the other cases due to local decoupling of cells when gap junction conductance is decreased. This resulted in an S_V_ that is 87% larger than baseline and 46% larger than with 50% I_K1_ alone.

These results show that the filtering method accurately estimates fluctuations in V_max_ based on the J_RyR_ profile. Moreover, the new empirical relationships derived here between J_RyR_ and V_max_ provide an approach to yield new insight and intuition into how changes in cellular properties and environment, such as reductions in outward currents and electrotonic coupling analyzed here, alter and in this case enhance spatial fluctuations in V_max_.

### Resampling method for estimating rare event probabilities

**Fig 10:**
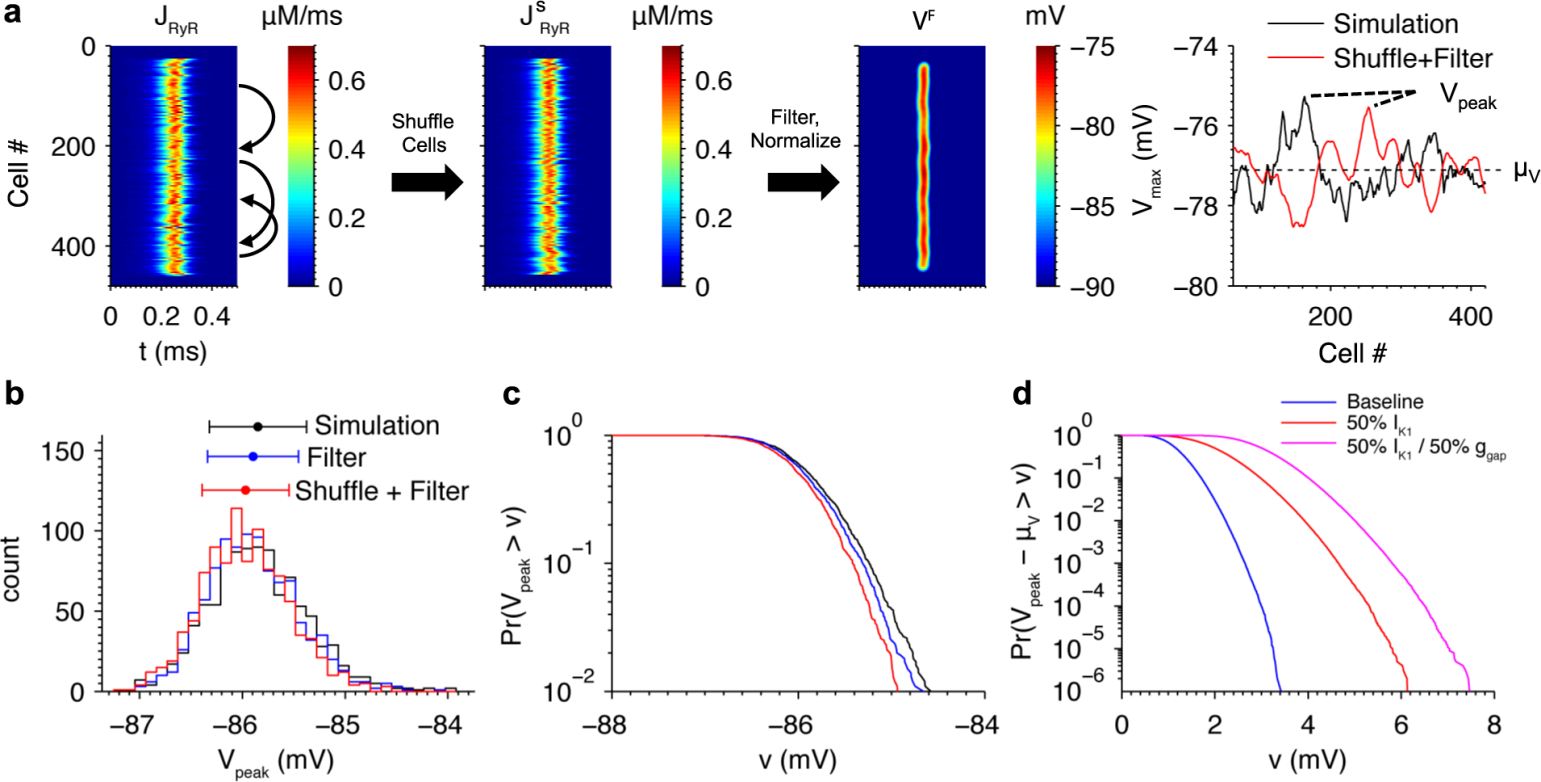
Method for estimation of rare extreme DAD probabilities. (a) Example illustrating how independent fiber realizations are generated by shuffling cell positions and applying the filtering method. The resulting V_max_ profile of the original simulation (black) and V^f^_max_ obtained after shuffling (red) are plotted on the right. V_peak_ is defined as the maximum potential achieved along the length of the fiber. (b) Histograms of V_peak_ from model simulations (black), the filtering-only method (blue), and filtering and shuffling method (red). See text for details. (c) Upper tail of the distributions from (b). (d) Predicted tail of V_peak_ distributions plotted relative to μ_V_ for fibers with the baseline model (blue), with 50% I_K1_ reduction (red), and with both 50% I_K1_ reduction and 50% g_gap_ (magenta).

The filtering method enables studies of properties of V_max_ without the need to simulate the full biophysical model. Studying the statistical properties of V_max_ requires the generation of large numbers of realizations. To do this, independent samples of V_max_ were generated by shuffling cell positions in the J_RyR_ profile, as illustrated in Fig 10A. Note that boundary effects the outer 24 cells on either end of the fiber are initialized to normal SR Ca^2+^ loads and are not included in the shuffle. The filter method was then applied to the shuffled J_RyR_ profile to estimate V_max_. An example V_max_ profile obtained using this method is shown on the right. Note that the fluctuations about the mean μ_V_ are qualitatively similar to those of the original simulation. In Fig 7, it was shown how a triggered propagating ectopic beat originated from a region of the fiber that experienced extreme DADs. Unfortunately it is impossible to perform the millions of cable simulations using the full biophysical model that are needed to study the probability of generating ectopic beats as model parameters are varied. However, our shuffling and filtering method does enable us to study the probability of a surrogate event of interest for each fiber simulation, with the surrogate event defined as the greatest value of V_max_ realized along the fiber, referred to here as V_peak_.

The shuffling of cell positions makes two assumptions. The first is that the J_RyR_ profiles of the cells are decoupled stochastic processes. The second assumption is that the fiber contains a sufficient number of cells such that the true distribution of J_RyR_ is well represented by the collection obtained from a single simulation.

Previous work has shown that sub-threshold membrane depolarization induces Ca^2+^ sparks and waves [56]. It is unclear whether local depolarizations in the membrane potential caused by spontaneous release affects release in neighboring cells, which would invalidate the independence assumption. This was tested by computing the peak amplitude and peak time of [Ca^2+^]_i_ in the fiber simulation represented by the red trace in Fig 8C. The absolute difference in the peak amplitude and peak time were then computed for all adjacent cell pairs and all cell pairs separated by a 50-cell distance along the fiber. Assuming that Ca^2+^ release in 50^th^ neighbors are decoupled, if release in adjacent cells were coupled then one would expect the distributions of peak amplitude and timing to differ from those of the 50^th^ neighbor pairs. In particular, if local Ca^2+^ release events were more synchronized, one would expect the difference in timing to be smaller. However, no substantial differences in Ca^2+^ release peak or timing were observed (S4 Fig), as these distributions also were not significantly different according to non-parametric Kruskal-Wallis tests (p = 0.77, 0.66). Therefore the coupling effect of adjacent cells does not substantially affect spontaneous Ca^2+^ release, which can thus be treated as a decoupled process in each cell in the fiber.

Validation of these assumptions also requires that the distribution of V_peak_ generated using this method match that obtained when using the detailed biophysical model. This distribution was estimated from 1,000 independent realizations. Simulating the ~500-cell fiber with 25,000 release sites in each cell is computationally prohibitive. For the purposes of the validation, the number of release sites was reduced to 2,500 and the fiber length to 96 cells. The values of μ_V_ and σ_V_ were computed using the V_max_ values from all cells in all fibers.

Fig 10B compares the true distribution of V_peak_ computed using the full biophysical model to that obtained from applying only the filtering method to the simulated J_RyR_ profiles (without shuffling). While this distribution exhibits a very small bias of ~ -0.05 mV, it is not significantly different from the true distribution on the basis of a Kruskal-Wallis test after correcting for the bias by subtracting their means (p = 0.89). The shuffling method was then validated by resampling from the J_RyR_s in all 1,000 fibers to produce 1,000 96-cell fiber realizations and computing V_peak_ with the filtering method. While the resulting distribution also exhibits a very small bias of ~ -0.08 mV, it is not significantly different from the true distribution after adjusting the means (p = 0.69) (Fig 10B). The upper tails of the distributions containing the extreme DADs of interest are also similar (Fig 10C). To validate the second assumption that the sampling population of J_RyR_ is sufficiently large, these tests were repeated using a subset of 5 of the 1,000 fibers to compute μ_V_, σ_V_, and the population of resampled J_RyR_’s (bias +0.05 mV, p = 0.68). Therefore, the method is accurate using a total of ~ 500 simulated cells. In summary, the method outlined here is a computationally efficient approach that permits the rapid estimation of the V_peak_ distribution using output from a single 500-cell fiber simulation.

### Prediction of rare events

Fig 10D plots the probability that V_peak_—μ_V_ exceeds a given potential estimated from an ensemble of 10^6^ realizations. Reducing I_K1_ by 50% shifts the distribution tail to greater amplitude DADs compared to the baseline model. The probability of observing a V_peak_ 3 mV greater than μ_V_ (-77.1 mV) is 9.9 × 10^−5^ in the baseline model compared to 0.096 with 50% reduction in I_K1_, a nearly 1000-fold increase. The most extreme event observed with 50% I_K1_ was 6.1 mV greater than μ_V_ compared to 3.4 mV in baseline, corresponding to DAD amplitudes 41% and 21% above average, respectively. Reducing g_gap_ by 50% further increases the likelihood of large-amplitude DADs, with the probability of a 3 mV DAD reaching 0.50, a 5000-fold increase over baseline, and the most extreme event observed at 7.5 mV, corresponding to a DAD amplitude 53% higher than average. These results demonstrate how changes ion channel expression or function can alter the probability of supra-threshold membrane potential fluctuations. In this case, reductions in I_K1_ and g_gap_ considerably widen the distribution of V_peak_ and increase the probability of occurrence of larger V_peak_ values by multiple orders of magnitude.

**Fig 11:**
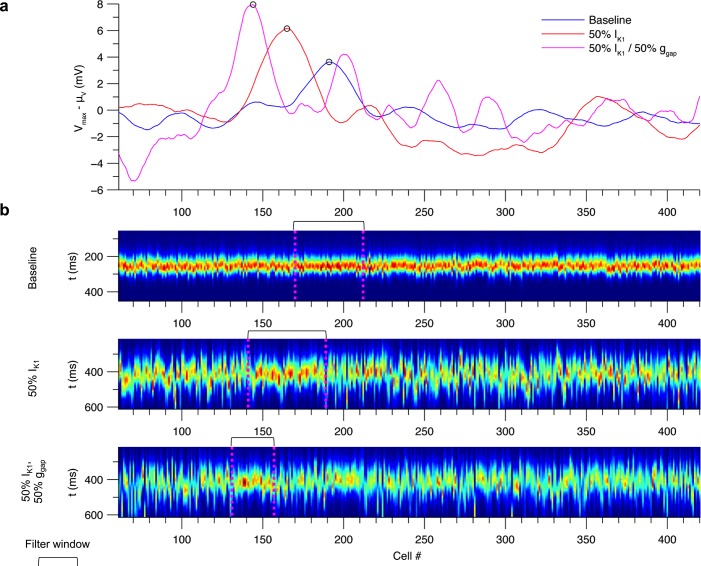
Realizations of rare extreme DADs. (a) Plots of V_max_—μ_V_ in the realizations from Fig 10D containing the most extreme DADs out of 10^6^ trials. Curves correspond to the baseline model (blue), 50% reduction of I_K1_ (red), and both 50% I_K1_ and 50% g_gap_ (magenta). (b) Spatiotemporal J_RyR_ profiles in each realization from panel (a). Brackets and dotted lines mark the location of the filter window centered on the extreme DAD.

To illustrate the nature of these rare events, the realizations exhibiting the greatest V_peak_ values were examined in each condition. Fig 11A plots V_max_—μ_V_. Because V_max_ at any position is determined by an average of the Ca^2+^ release profiles over a window of cells, each rare depolarization occurs at a cluster of cells whose width corresponds to the window size. Fig 11B shows the underlying J_RyR_ profiles and the filter window centered on the V_peak_. In each case, the extreme event occurs at a cluster of cells within the window where J_RyR_ tends to be greater and more synchronized than in the rest of the fiber. This result demonstrate that rare (occurring ~1 in 10^6^ beats in a 500-cell fiber) proarrhythmic events result from spontaneous Ca^2+^ release exhibiting high (1) flux magnitude, (2) synchronization, and (3) spatial clustering. None of the 999,999 other simulations result in such an extreme event due to a lack of one or more of these properties. Note that because the window size is smaller in the case of 50% g_gap_, fewer cells need to fulfill these three criteria and thus the probability of extreme events is much higher.

## Discussion

In this study we have presented a biophysically detailed stochastic computational model of the ventricular cardiac myocyte describing spatial Ca^2+^ diffusion between release sites and incorporated it into a tissue-scale model to study the mechanisms and statistical properties of DADs. Loading of SR Ca^2+^ is known to cause spontaneous Ca^2+^ release [6, 47, 57]. In this model, Ca^2+^ waves were generated when passive diffusion of Ca^2+^ between release sites caused Ca^2+^ sparks to propagate across the cell. In agreement with experimental studies, RyR sensitivity modulated the threshold SR Ca^2+^ load at which this instability arises [58]. Rather than explicitly modeling RyR regulation mechanisms such as interaction with calmodulin [59], phosphorylation by activated CaMKII [60] and PKA [61], allosteric channel decoupling due to PKA-dependent dissociation of FKBP12.6 [62], and oxidation by reactive oxygen species [33, 41], RyR opening rate was scaled such that the model reproduced experimentally observed triggered APs at 1 Hz pacing [33]. [Ca^2+^]_JSR_-dependent regulation of the RyRs increases their sensitivity to cytosolic [Ca^2+^] [63, 64], but its role in spontaneous Ca^2+^ release is controversial. In our model of RyR gating, it was assumed that this mechanism has little effect on RyR open probability when [Ca^2+^]_JSR_ < 1 mM [65, 66] and therefore played a negligible role in dynamically regulating the RyRs in this study.

In our investigation, we found that pacing the baseline model at 1 Hz with 50% I_K1_ inhibition (in the absence of β-adrenergic stimulation) caused increased APD and cellular Ca^2+^ loading over time. This resulted in greater inward NCX current during the repolarization phase, thus further increasing APD and Ca^2+^ loading. This positive-feedback loop led to EADs and ultimately failure to repolarize. It is possible that the saturating behavior of LCC Ca^2+^-dependent activation in this model prevents sufficient LCC inhibition required for repolarization in the presence of elevated SR Ca^2+^. The LCC model also does not incorporate “global” [Ca^2+^]_i_ sensing [67], which would inhibit LCC openings in the presence of sustained elevated [Ca^2+^]_i_. Future work is needed to understand the contributions of these mechanisms, as they may affect the conditions under which the model exhibits DADs during pacing.

The distribution of DADs was controlled by both [Ca^2+^]_i_ and [Ca^2+^]_SR_. This was revealed by the apparent change in the threshold SR Ca^2+^ load for spontaneous Ca^2+^ wave formation during pacing (see Fig 3). Elevated diastolic [Ca^2+^]_i_ increased RyR opening rate and thus perpetuated Ca^2+^ wave formation at lower SR Ca^2+^ loads. It also caused more rapid loading of the SR to induce overload. Cellular Ca^2+^ loading also increased the amplitude of DADs due to the greater number and concurrence of Ca^2+^ wave nucleation sites, which is in agreement with experimental studies [9, 10]. Consistent with our results, Wasserstrom et al. reported that cellular Ca^2+^ loading reduced DAD delay and increased synchrony associated this with greater likelihood of triggered activity [10].

The formation of Ca^2+^ sparks and waves may be influenced by additional factors not considered in this study. These include intrinsic properties such as release site heterogeneity, including dyad geometry, RyR cluster morphology [19, 68], local SR connectivity [69], and release site spacing [70, 71]. Additionally, cell-to-cell heterogeneity would also affect DADs in tissue. Further work is needed to quantify the contributions of these factors to DAD variability.

Triggered events were investigated using a fiber model. Experimental study of the nature of DAD-induced arrhythmias is difficult due the limited temporal and spatial resolutions of live multiplex fluorescence imaging of tissue preparations. Xie et al. used a computational tissue model to study how the critical size of an isolated cluster of cells exhibiting identical spontaneous Ca^2+^ release events required to trigger an AP depended on a variety of tissue geometries and was reduced under pathophysiological conditions [72]. They further showed that DAD amplitude and the critical cell mass depended sensitively on the amplitude of the spontaneous [Ca^2+^]_i_ transient. Here we have investigated stochastic variability in DAD amplitude in a long fiber of Ca^2+^-overloaded cells. We showed that large DADs occurred probabilistically due to random patterns of RyR gating and the formation of Ca^2+^ waves that gave rise to high-amplitude, synchronized Ca^2+^ release flux in a cluster of cells. Such events trigger arrhythmias by causing ectopic beats, inducing regional conduction block via Na^+^ channel inactivation [73], and increasing variability of repolarization [46]. Heterogeneity of cell types and intercellular variability of Ca^2+^ handling may also play an important role in determining the likelihood and location of triggered foci [10] but exploration of these factors remains beyond the scope of this study. Furthermore, the potential effects of pro-arrhythmic beat-to-beat AP variability [74] or EADs [75], which could affect Ca^2+^ loading and DAD timing, were not examined.

The role of I_K1_, which acts to stabilize the resting membrane potential, affected the DAD distribution in isolated cells. Loss of I_K1_ function has been associated with arrhythmogenesis in diseases such as heart failure [49], Andersen’s syndrome [50], and long QT syndrome [51]. Maruyama et al. showed that the Ca^2+^-membrane voltage gain, defined as the ratio of DAD amplitude to spontaneous [Ca^2+^]_i_ transient amplitude, increased following I_K1_ suppression [76]. Consistent with this finding, the model exhibited a considerable increase in DAD amplitude in both isolated cells and in the fiber when I_K1_ density was reduced by 50%. Most notably, I_K1_ suppression increased DAD amplitude variability, dramatically increasing (by 1000-fold) the probability of occurrence of potentially arrhythmogenic large-amplitude DADs. Note that the model does not include Ca^2+^-dependent regulation of I_K1_, as this mechanism has been controversial [77–79]. While a recent study by Nagy et al. [80] suggests elevating [Ca^2+^]_i_ at a fixed concentration upregulates I_K1_ via an increase in CaMKII activity, there remains insufficient data to constrain a model of this mechanism. Future models could be informed by further studying dynamic changes in CaMKII activity and I_K1_ in response to elevated [Ca^2+^]_i_. Reduction of gap junction conductance, another pathological feature of diseases such as HF [55], further increased the variability of DAD amplitude in the fiber by reducing the spatial scale of electrotonic coupling. These findings are consistent with a previous modeling study that showed that fewer contiguous cells are required to exhibit DADs to produce a triggered beat under such conditions [72].

The relationship between I_K1_ and/or g_gap_ to the probability of occurrence of extreme DADs demonstrated here is but one of many possible examples of how alterations of cellular mechanisms or environment can modulate propensity for arrhythmia. For example, HF also causes extensive remodeling of the TTs and dyads, leading to reduced CICR efficiency [81, 82]. The model can be used to directly probe the effects of corresponding perturbations such as the number of release sites, RyR cluster size, and dyad volume on intracellular Ca^2+^ cycling, the formation of spontaneous Ca^2+^ sparks and waves, and rare arrhythmic events. Thus the novel method presented here provides a general framework for prediction of arrhythmia likelihoods in response to specific aspects of disease-related physiological remodeling.

A significant contribution of this work is that the emergence of sudden arrhythmias can be causally linked to stochastic molecular events. A computationally efficient method was developed to estimate the probability of extreme DADs. An important assumption of this method is that the spontaneous Ca^2+^ release events in neighboring cells are decoupled. It is known that membrane depolarization increases the frequency of Ca^2+^ waves by reducing NCX-mediated Ca^2+^ efflux and thus promoting Ca^2+^ waves due to increased intracellular [Ca^2+^] [56]. Therefore, a release event in one cell that causes a local increase in V may hasten spontaneous Ca^2+^ release in its neighbors. Validation of our method suggests that this coupling phenomenon has negligible impact on the V_max_ distribution because the effect is weak compared to the intrinsic variability of spontaneous Ca^2+^ release. Note that the model does not account for gap junction mediated intercellular Ca^2+^ diffusion, resulting in cell-to-cell transfer of Ca^2+^ waves, though the prevalence of such events remains unclear [9, 83–87].

There are two important conclusions that emerge from using this method. First, variability of the inward current due to stochastic RyR gating causes random patterns of Ca^2+^ wave dynamics and results in substantial DAD variability at the tissue scale, particularly in the pathological states tested where I_K1_ and g_gap_ were reduced. Second, while one can imagine a case where a contiguous cluster of cells exhibit large synchronized spontaneous Ca^2+^ release, the probability distribution of such events has not been well characterized. In a 496-cell fiber with reduced I_K1_ and gap junction coupling, the largest DAD amplitude observed in an ensemble of 10^6^ realizations had amplitude ~50% greater than the mean DAD amplitude. For such a fiber paced at 1 Hz and exhibiting a DAD after every beat, one could expect to observe such an event approximately once every 11 days. Thus extreme DADs, while quite rare, are still possible over relevant time frames. Further work is needed to estimate the probability of such events in whole heart, as 3D tissue is ~1–2 orders of magnitude less likely to exhibit triggered beats due to the increased electrotonic coupling [72] but also contain a much greater number of cells than tested here. Nevertheless, the results presented here suggest that variability due to stochastic molecular events play a large role in the initiation of cardiac arrhythmias and sudden cardiac death.

## Supporting information

S1 Fig NCX current during an action potential at 1 Hz pacing under normal conditions. (EPS)

S2 Fig Baseline model paced at 1 Hz for 16 seconds. The (a) action potential and (b, c) Ca^2+^ dynamics reach steady state after ~ 10 seconds.
(EPS)

S3 Fig Extended version of protocol in Fig 3. (a) DADs and triggered APs continue to occur after cessation of pacing after t = 30 s. [Na^+^]_i_ is ramped down to 15 mM over t = 35 to 40 s, resulting in sub-threshold DADs that occur at lower frequency. (b) APD of paced and DAD-triggered APs. APD decreases mainly as [Na^+^]_i_ rises due to reduced inward NCX current. (c) [Ca^2+^]_i_ and (d) average [Ca^2+^]_JSR_ are shown.
(EPS)

S4 Fig Independence of spontaneous Ca^2+^ release in a fiber. [Ca^2+^]_i_ profiles were taken from the simulation represented by the red trace in Fig 8C, a 496-cell fiber with 50% I_K1_ and 50% g_gap_. The absolute difference in peak [Ca^2+^]_i_ was computed for each pair of adjacent (dC_1_) and 50^th^ neighbor (dC_50_) cells. The absolute difference in the times of the peaks was also computed (dT_1_, dT_50_). Histograms and QQ plots of the differences in (a) time of peak and (b) peak [Ca^2+^]_i_. Both QQ plots exhibit linear trends, implying that the distributions are indeed alike. Therefore, the timing and amplitude of spontaneous Ca^2+^ release of adjacent cells do not differ substantially from those of distant cells.
(EPS)

S1 Equations Release site Ca^2+^ transport equations. (DOCX)

S1 Table Release site Ca^2+^ transport parameters. (DOCX)

S1 Text Supporting description of model and filtering method. (DOCX)

S1 Movie Volume rendering of single-cell spontaneous Ca^2+^ release. This illustrates the effect of varying SR Ca^2+^ loads on Ca^2+^ wave dynamics, as shown in Fig 4.
(M4V)

S2 Movie Volume rendering of nine independent single-cell simulations. Each was started with identical initial conditions to illustrate the variability in Ca^2+^ wave dynamics due to stochastic Ca^2+^ spark activity, as shown in Fig 5.
(M4V)
