# Supplementary material for: Estimating the probabilities of rare arrhythmic events in multiscale computational models of cardiac cells and tissue
Source: PLoS Comput Biol. 2017 Nov 16;13(11):e1005783. doi: 10.1371/journal.pcbi.1005783 (PMC5689829; doi:10.1371/journal.pcbi.1005783)
Supplement: S1 Equations — (DOCX) [file pcbi.1005783.s005.docx]

**S1 Equations.**

|  | . | (S1) |
| --- | --- | --- |
|  |  | (S2) |
|  |  | (S3) |
|  |  | (S4) |
|  |  | (S5) |
|  |  | (S6) |
|  |  | (S7) |
|  |  | (S8) |
|  |  | (S9) |
|  |  | (S10) |
|  |  | (S11) |
|  |  | (S12) |
|  |  | (S13) |
