# Supplementary material for: Estimating the probabilities of rare arrhythmic events in multiscale computational models of cardiac cells and tissue
Source: PLoS Comput Biol. 2017 Nov 16;13(11):e1005783. doi: 10.1371/journal.pcbi.1005783 (PMC5689829; doi:10.1371/journal.pcbi.1005783)
Supplement: S1 Table — (DOCX) [file pcbi.1005783.s006.docx]

**S1 Table. Release site Ca^2+^ transport parameters.**

| V_JSR_ | JSR volume | 1.113 × 10^-11^ μL |
| --- | --- | --- |
| V­_SS_ | Subspace volume | 0.812 × 10^-12^ μL |
| V_SM_ | Sub-membrane volume | 5.85 × 10^-11^ μL |
| B_csqn_ | Calsequestrin buffer concentration | 13.5 mM |
| K_csqn_ | Calsequestrin buffer affinity | 0.63 mM |
| B_SR_ | SR membrane buffer site concentration | 0.047 mM |
| K_SR_ | SR membrane buffer site affinity | 0.00087 mM |
| B_SL,SS_ | Sarcolemmal membrane buffer site concentration (subspace) | 1.124 mM |
| B_SL,SM_ | Sarcolemmal membrane buffer site concentration (sub-membrane) | 0.122 mM |
| K_SL_ | Sarcolemmal membrane buffer site affinity | 0.0087 mM |
| B_cmdn_ | Calmodulin buffer concentration | 0.05 mM |
| K_cmdn_ | Calmodulin buffer affinity | 2.38 × 10^-3^ mM |
| v_RyR_ | Open RyR Ca^2+^ transport rate | 0.92 ms^-1^ |
| P_CaL_ | LCC permeability | 9.13 × 10^-13^ cm μF s^-1^ |
| τ_NSR-JSR_ | NSR-JSR Ca^2+^ diffusion time constant | 9 ms |
| τ_SS-SM_ | SS-SM Ca^2+^ diffusion time constant | 0.02 ms |
| τ_SM-Cyto_ | SM-Cyto Ca^2+^ diffusion time constant | 1 ms |
| τ_Long_ | Longitudinal Ca^2+^ diffusion time constant | 4 ms |
| τ_Trans_ | Transverse Ca^2+^ diffusion time constant | 2 ms |
