# Supplementary material for: Estimating the probabilities of rare arrhythmic events in multiscale computational models of cardiac cells and tissue
Source: PLoS Comput Biol. 2017 Nov 16;13(11):e1005783. doi: 10.1371/journal.pcbi.1005783 (PMC5689829; doi:10.1371/journal.pcbi.1005783)
Supplement: S1 Text — (DOCX) [file pcbi.1005783.s007.docx]

**Supporting Information**

**S1 Text. Supporting description of model and filtering method.**

**LCC Markov Model.** The model incorporates the LCC Markov model described by Greenstein and Winslow. In the baseline model, it was assumed that 25% of LCCs were functionally active, which were randomly selected at the beginning of each simulation. The function *γ* that controls the rate into the Ca^2+^ -inactivated states was changed from a linear to a saturating function of [Ca^2+^]_d,i,j,k_ (in mM):

|  | . | (S1) |
| --- | --- | --- |

**Sensitivity Analysis of the Filtering Method.** According to Eq. (6), the ratio σ_V_/σ_J_ represents the sensitivity of V_max_ with respect to deviations from the average in the filtered release flux values, J`_max_-μ_J_. Compared to the baseline, the sensitivity was 46% larger with 50% I_K1_ density and 17% larger with both 50% I_K1_ and 50% g_gap_ due to the reduced outward current. In the latter case, the sensitivity was 20% lower than with 50% I_K1_ alone. This result is counter-intuitive, as one would expect the sensitivity of V_max_ with respect to J_RyR_ to increase due to the reduced electrotonic load.

This discrepancy can be explained by the fact that the filter width was reduced by ~50% after introducing 50% g_gap_. Note that the estimate of V is given by,

|  | . | (S2) |
| --- | --- | --- |

Combining Eqs. (4) and (S15) yields the expression

|  | , | (S3) |
| --- | --- | --- |

where N is the number of cells in the fiber. Note the summations in second term in parentheses can be exchanged and simplified to yield

|  | . | (S4) |
| --- | --- | --- |

The partial derivative of V` at cell x with respect to J`_RyR_ of any cell x-(W-1)/2 ≤ x` ≤ x+(W-1)/2, lying within the filter window centered on x, is therefore approximated by

|  | , | (S5) |
| --- | --- | --- |

assuming that the filter width is much smaller than the fiber length (W/N << 1). Therefore V` is approximated by the linear function

|  | , | (S6) |
| --- | --- | --- |

The variance of V` therefore given by

|  | . | (S7) |
| --- | --- | --- |

Combining Eqs. (S6) and (S7) above and applying the linearity property of expectation gives the result

|  | . | (S8) |
| --- | --- | --- |

Therefore the standard deviation of V` is given by the product of (σ_V_/σ_J_)/W and the standard deviation of J_RyR_ over the filter window.
